# Microarray analysis reveals the potential molecular mechanism of Lp299v in stable coronary atherosclerotic disease

**DOI:** 10.1186/s13568-022-01466-y

**Published:** 2022-09-24

**Authors:** Zhenyang Fu, Xiaolei Song, Anna Shen, Tao Zhou

**Affiliations:** 1 Department of Cardiology, The Third Affiliated Hospital of Southern Medical University, Southern Medical University, Guangzhou, 510630 China; 2 Department of Obstetrics and Gynecology, The Third Affiliated Hospital of Guangzhou Medical University,Guangzhou Medical University, Guangzhou, China

**Keywords:** Coronary atherosclerotic disease, *Lactobacillus plantarum* 299v, Microarray analysis, Immune response, G protein-coupled receptor

## Abstract

A growing body of evidence has confirmed that inflammatory mechanisms are involved in the formation and treatment of coronary atherosclerotic disease (CAD). An increase in circulatory levels of inflammatory cytokines has been found in patients with CAD, while the molecular mechanisms of inflammation still remain elusive. This study was designed to identify differentially expressed genes (DEGs), and to explore the molecular mechanism and hub genes that are involved in the effects of *Lactobacillus plantarum* 299v (Lp299v) supplementation. Microarray dataset (GSE156357) was downloaded from the Gene Expression Omnibus (GEO) database. The DEGs were identified by the R software. Then, the Gene Ontology (GO) and the Kyoto Encyclopedia of Genes and Genomes (KEGG) pathway enrichment analyses and construction of protein–protein interaction (PPI) network were performed by DAVID, STRING, and Cytoscape software. In daily alcohol user (DAU) group, 7,541 DEGs were identified, including 206 up-regulated and 7,335 down-regulated DEGs. In non-daily alcohol user (non-DAU) group, 2,799 DEGs were identified (2,491 up-regulated and 308 down-regulated DEGs). The GO enrichment analysis revealed that miosis was up-regulated and immune response was down-regulated. The KEGG enrichment analysis showed that Lp299v supplementation reduced the levels of chemotactic cytokines, and weakened immune response. Proteins of G protein-coupled receptor, inflammatory response, regulation of cell proliferation and apoptosis-related proteins were found in the PPI network. The hub genes were associated with G protein-coupled receptor, inflammatory response, and cell proliferation and apoptosis. The weighted gene co-expression network analysis (WGCNA) enriched the DEGs in 4 modules. This study indicated the expressions of chemokine receptors and regulation of immune response in the Lp299v supplementation. Meanwhile, it was supposed that chemokine receptors may have a cellular effect.

**Supplementary Information:**

The online version contains supplementary material available at 10.1186/s13568-022-01466-y.

## Introduction

Traditionally, the pathophysiology of coronary atherosclerotic disease (CAD) has been identified in the formation of coronary atherosclerotic plaques, which can cause chronic narrowing of coronary lumen, and may even cause rupture of coronary atherosclerotic plaques and thrombosis, eventually leading to acute myocardial infarction. An increasing number of studies have confirmed that inflammatory mechanisms are not only involved in the formation of CAD, but also affect the treatment and prognosis of patients with CAD (Garofallo et al. 2019; Libby et al. 2005; Pugliese et al. 2020). The levels of C-reactive protein and IL-6 were reduced in patients with stable CAD who took statins compared with those who underwent elective coronary stent implantation and did not take statins, suggesting an association between CAP and inflammatory response (Moratalla et al. 2016). The increase in the concentrations of fibrinogen and C-reactive protein causes an elevation in acute phase proteins and cytokines, such as TNF and IL-6, which further activates monocytes/macrophages and T cells, and leads to atherosclerosis (Schaberg et al. 1992; Tappia et al. 1995). Macrophages and T lymphocytes are thought to play an important role in the formation of coronary atherosclerotic plaques (Hedrick 2015; Moore et al. 2018).

Several studies have shown that gut microbiota play a significant role in CAD, heart failure, and metabolic disorders (Brial et al. 2018; Kitai et al. 2018; Tang et al. 2017). The influences of gut microbiota on these diseases may be associated with regulation of intestinal or systemic inflammation. *Lactobacillus plantarum* 299v (Lp299v), a member of the Lactobacillus family, is widely found in dairy products, meat, and fermented vegetables. Lp299v has a significant antioxidant activity, and it can inhibit intestinal bacteria and intestinal inflammations (Bested et al. 2013; Bixquert Jimenez 2009). A prospective study demonstrated that Lp299v improved vascular endothelial function and reduced systemic inflammation in patients with stable CAD (Malik et al. 2018). In a controlled, randomized, double-blinded study, the experimental group who drank beverages containing Lp299v had significantly greater systolic blood pressure, leptin, and fibrinogen than those in the control group, in which a 37% reduction in F2-isoprostaglandin levels and a 42% reduction in IL-6 levels were observed as well (Naruszewicz et al. 2002). Subsequent studies confirmed that Lp299v possessed a systemic anti-inflammatory effect and reduced the activity of circulatory inflammatory markers, and it was found that the levels of Janus kinase 2, Guanylate-binding protein 1, and TNF superfamily member 10 were reduced in blood after oral Lp299v supplementation (Hofeld et al. 2021).

Hence, the present study aimed to identify differentially expressed genes (DEGs) and the hub genes, so as to explore the molecular mechanism. In this study, microarray datasets from the Gene Expression Omnibus (GEO) database were downloaded and analyzed to obtain DEGs, and serum levels were measured before and after oral Lp299v in daily alcohol user (DAU) and non-DAU patients with stable CAD (Hofeld et al. 2021). Subsequently, the Gene Ontology (GO) and the Kyoto Encyclopedia of Genes and Genomes (KEGG) pathway enrichment analyses and construction of a protein–protein interaction (PPI) network were performed to investigate the molecular mechanisms of Lp299v supplementation in patients with stable CAD.

## Methods

### Microarray datasets

The microarray dataset (GSE156357) was downloaded from the GEO database (https://www.ncbi.nlm.nih.gov/gds). The probes were converted into the corresponding gene symbols according to the annotation information in the platform. The GSE156357 dataset included 19 pre-supply samples and 19 post-supply samples. Plasma samples obtained from these patients before and 6 weeks after Lp299v supplementation were stimulated with peripheral blood mononuclear cells from healthy donors, which were sequenced to obtain raw data. A total of 38 samples were obtained from 19 patients before and after oral Lp299v supplementation (Hofeld et al. 2021).

### Identification of DEGs

The DEGs of pre-supply and post-supply samples were screened using R software in the two groups. Benjamini–Hochberg adjusted P was used to control false discovery rate (FDR). Probe sets without corresponding gene symbols or genes with more than one probe set were removed or averaged. Gene expression levels of |logFC|> 0.3 and *P* < 0.05 were chosen as thresholds.

### GO and KEGG pathway enrichment analyses of DEGs

The GO and KEGG pathway enrichment analyses were performed by the Database for Annotation, Visualization and Integrated Discovery (DAVID, http://david.ncifcrf.gov) in two groups. *P* < 0.05 was considered statistically significant.

### PPI network construction and module analysis

The PPI network was constructed using the Search Tool for the Retrieval of Interacting Genes (STRING: http://string-db.org) to identify direct or indirect associations between proteins. The STRING does not support networks with higher than 2,000 nodes. The number of proteins was reduced by chosen thresholds of the gene expression levels of |logFC|> 0.7 and *P* < 0.05 in the DAU group, and |logFC|> 0.4 and *P* < 0.05 in the non-DAU group. A combined score > 0.9 was used to construct the PPI networks, which were visualized by Cytoscape software. The Molecular Complex Detection (MCODE) plug-in was used to select important functional modules of protein interaction networks.

### Selection of the hub genes in the network

The maximal clique centrality of each node was calculated by CytoHubba, a plug-in in Cytoscape, and the top 10 genes with the highest degree were regarded as hub genes in the PPI networks. Genes with the deepest color were considered as the hub genes in the network.

### Weighted gene co-expression network analysis (WGCNA)

The R software was used to remove outliers and samples, and WGCNA was additionally utilized to construct a scale-free co-expression network. First, Pearson correlation matrix and average linkage method were used for all paired genes, and then, the power function was utilized to construct the weighted adjacency matrix. The adjacency was transformed into a topological overlap matrix (TOM), and the corresponding dissimilarity was calculated. To classify genes with similar expression profiles into gene modules, average linkage hierarchical clustering was conducted according to the TOM-based dissimilarity measure.

## Results

### Identification of DEGs

After normalization, 7541 DEGs were identified in the DAU group (206 up-regulated and 7335 down-regulated DEGs). In the non-DAU group, 2799 DEGs were identified, including 2491 up-regulated and 308 down-regulated DEGs. A heatmap was plotted for each group.

**Fig.1:**
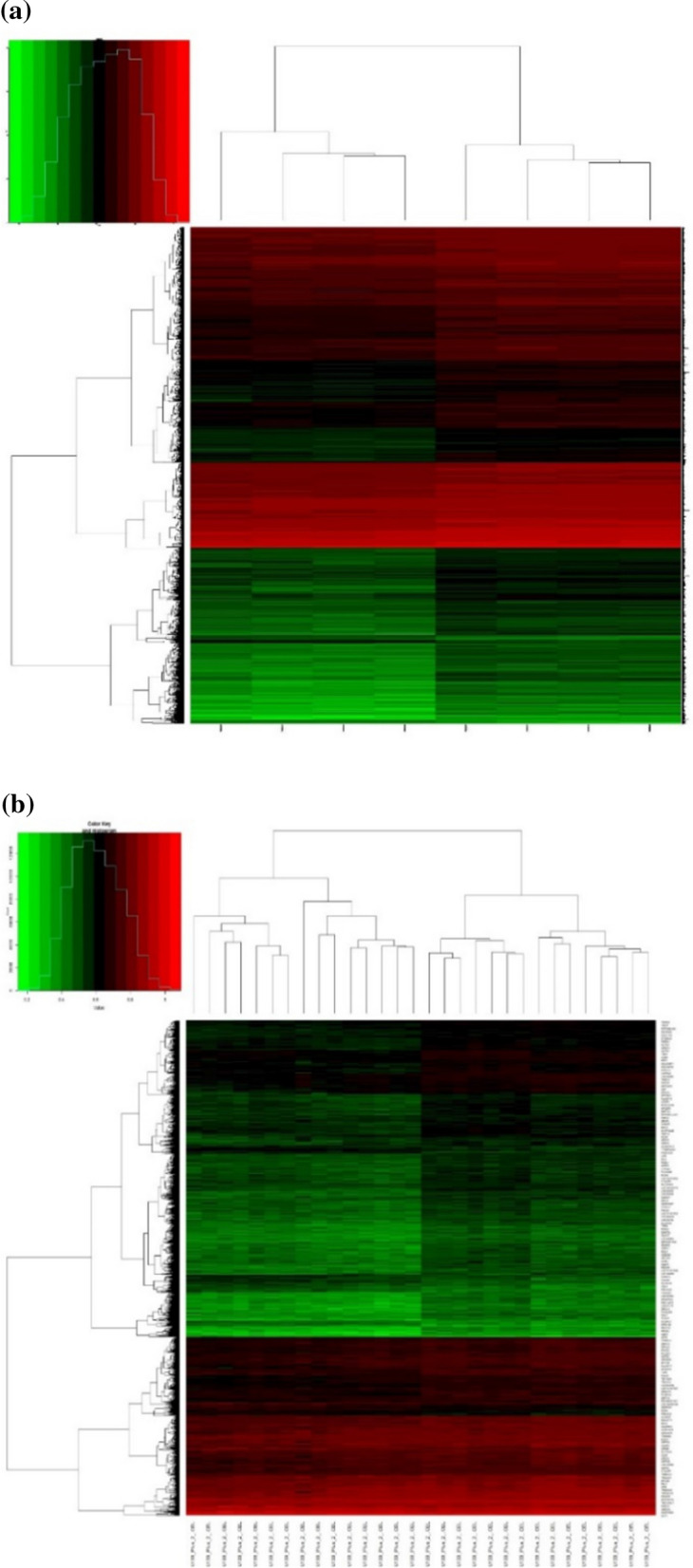
**a** In the daily alcohol user group, there are a total of 8 samples, 4 of which are the control group and 4 are the experimental group. After normalization, 206 up-regulated genes and 7335 down-regulated DEGs were found in the experimental group (*P* < 0.05). **b** There were 38 samples in the non-daily alcohol user group, of which 19 were the control group and 19 were the experimental group. We found 2491 up-regulated genes and 308 down-regulated DEGs in the experimental group (*P* < 0.05)

Using the two heatmaps, it was (Fig. 1) found that after Lp299v treatment, the differential gene expression levels in the treatment group were significantly changed compared with the control group.

### The GO and KEGG pathway enrichment analyses of DEGs

**Fig. 2:**
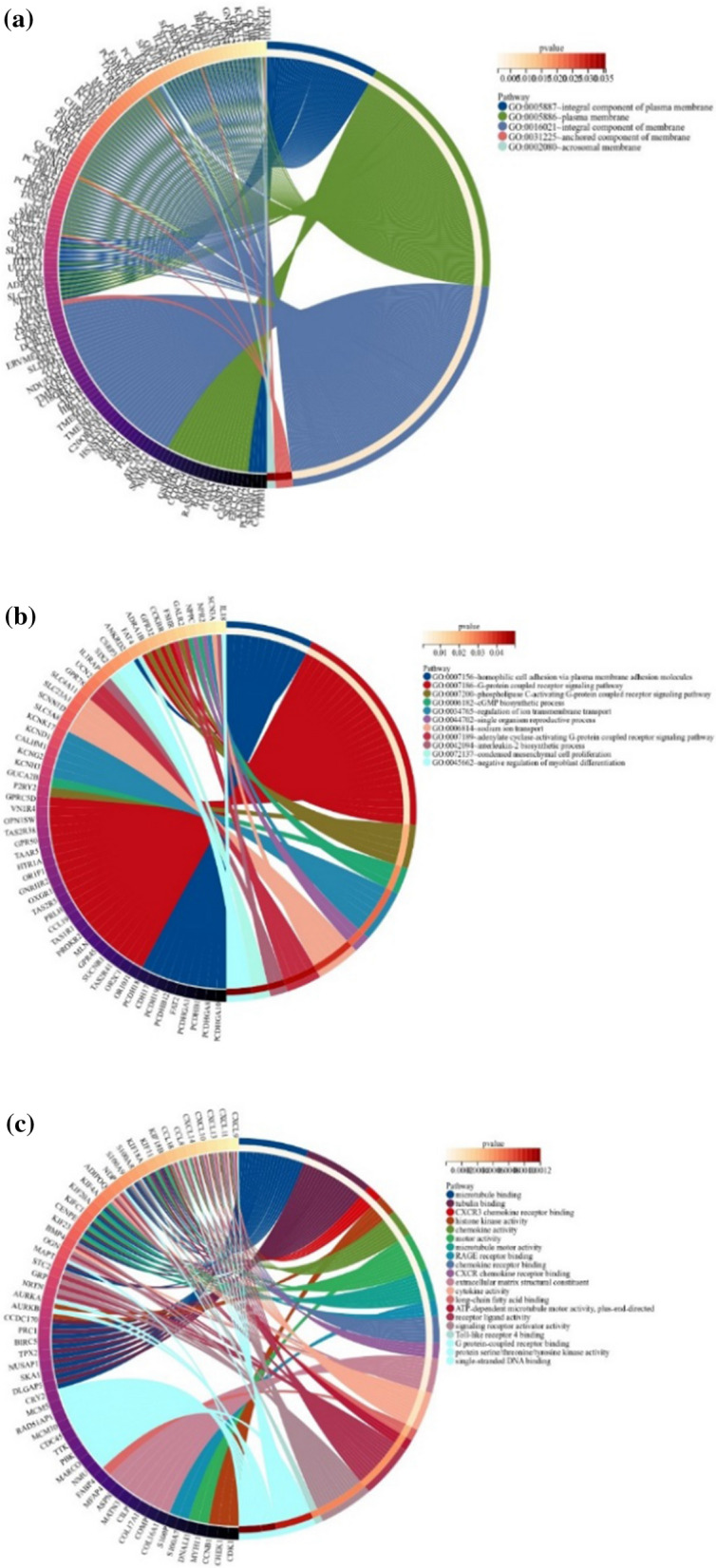
By performing GO enrichment analysis on DEGs, we obtain the circle maps of cell component, biological process, and molecular function. **a** The enriched cell component suggests changes in the composition of cell miosis component. **b** The enriched biological process suggests activation of G protein-coupled receptor-related signaling pathways and changes in ion transport. **c** The enriched molecular function suggested the involvement of various proteins and factors involved in the regulation of immune response, chemokines, etc.

As shown in Table 1 and Fig. 2a, changes in the cellular component (CC) of DEGs were mainly enriched in the miosis component. It is mainly reflected in the upregulation of cellular component pathways during cell mitosis.

**Table 1:** The top enriched cell component of DEGs in the daily alcohol user group group sorted by adjusted P-values in a descending order

Category	GOID	GO name	Count	Adjusted *P*-value	Regulation
CC	GO:0005819	Spindle	25	8.01E-23	Up
CC	GO:0072686	Mitotic spindle	16	2.03E-19	Up
CC	GO:0000775	Chromosome, centromeric region	18	4.99E-19	Up
CC	GO:0000779	Condensed chromosome, centromeric region	15	3.06E-18	Up
CC	GO:0005876	Spindle microtubule	12	1.79E-17	Up
CC	GO:0000776	Kinetochore	15	5.72E-16	Up
CC	GO:0098687	Chromosomal region	22	9.42E-16	Up
CC	GO:0000793	Condensed chromosome	18	3.30E-14	Up
CC	GO:0000777	Condensed chromosome Kinetochore	12	9.75E-14	Up
CC	GO:0030496	Midbody	13	2.10E-12	Up

As presented in Table 2 and Fig. 2b, changes in the biological process (BP) of DEGs were significantly enriched in the mitosis process. The specific performance is the biological process of organelle synthesis and regulation in cell mitosis.

**Table 2:** The top enriched biological process of DEGs in the daily alcohol user group sorted by adjusted P-values in a descending order

Category	GOID	GO name	Count	Adjust *P*-value	Regulation
BP	GO:0140014	Mitotic nuclear division	34	8.01E-23	Up
BP	GO:0000280	Nuclear division	36	2.03E-19	Up
BP	GO:0048285	Organelle fission	37	4.99E-19	Up
BP	GO:0000070	Mitotic sister chromatid segregation	24	3.06E-18	Up
BP	GO:0000819	Sister chromatid segregation	25	1.79E-17	Up
BP	GO:0007059	Chromosome segregation	29	5.72E-16	Up
BP	GO:1902850	Microtubule cytoskeleton organization involved in mitosis	21	9.42E-16	Up
BP	GO:0034765	Nuclear chromosome segregation	25	3.30E-14	Up
BP	GO:0031225	Mitotic spindle organization	18	9.75E-14	Up
BP	GO:0005179	Regulation of mitotic nuclear di-vision	19	2.10E-12	Up

As shown in Table 3 and Fig. 2c, changes in molecular function (MF) were mainly enriched in microtubule binding and immune response. We could imply the downregulation of MF in the immune response and the upregulation of MF in microtubule binding.

**Table 3:** The top enriched molecular function of DEGs in the daily alcohol user group sorted by adjusted P-values in a descending order

Category	GOID	GO name	Count	Adjusted *P*-value	Regulation
MF	GO:0008017	Microtubule binding	18	1.86E-07	Up
MF	GO:0015631	Tubulin binding	18	1.06E-05	Up
MF	GO:0048248	CXCR3chemokine receptor binding	4	1.06E-05	Down
MF	GO:0035173	Histone kinase activity	5	7.82E-05	Down
MF	GO:0008009	Chemokine activity	7	7.82E-05	Down
MF	GO:0003774	Motor activity	10	0.000107135	Up
MF	GO:0003777	Microtubule motor activity	8	0.000107135	Up
MF	GO:0050786	RAGE receptor binding	4	0.000249883	Down
MF	GO:0042379	Chemokine receptor binding	7	0.000504897	Down
MF	GO:0045236	CXCR chemokine receptor binding	4	0.001751271	Down

**Fig. 3:**
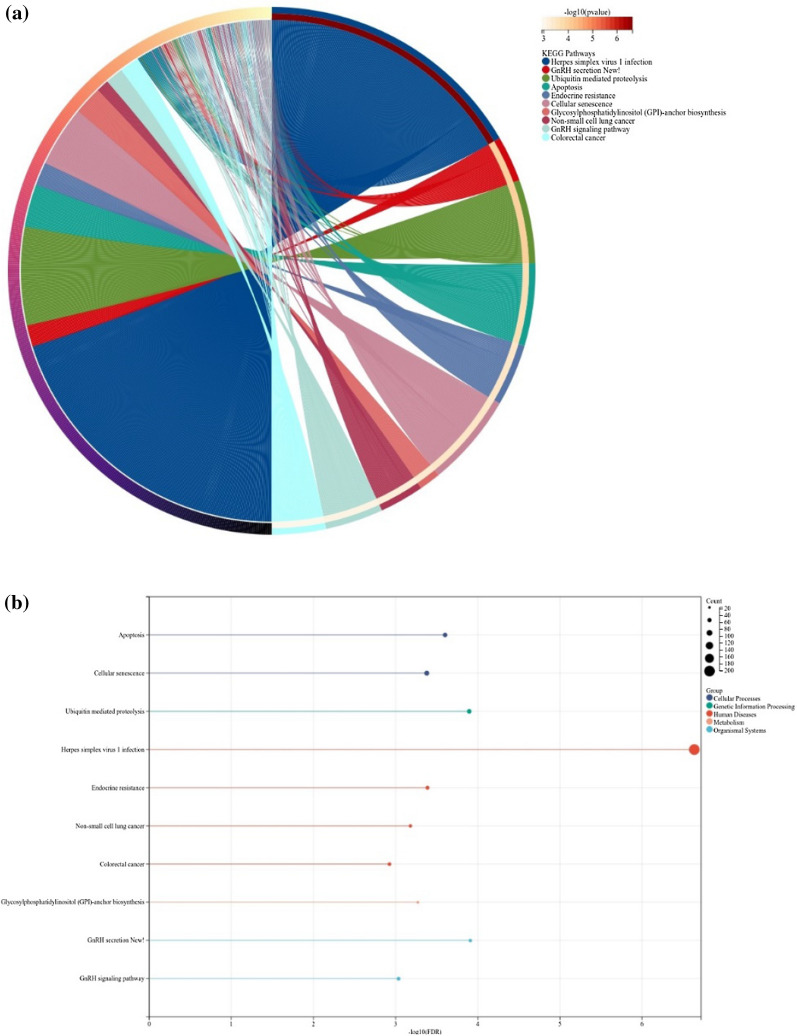
**a** Fig. 3a shows the chord diagram of the results of the KEGG pathway enrichment in the daily alcohol user group. In this figure, the following signaling pathways were enriched, herpes simplex virus 1 infection, gonadotropin-releasing hormone secretion, ubiquitin-mediated proteolysis, apoptosis, endocrine resistance, and cellular senescence. **b** Fig. 3b shows the histogram of the results of the KEGG pathway enrichment in the daily alcohol user group.

Although the number of DEGs in the DAU group (Fig. 3) was higher than that in the non-DAU group, according to the results of the GO enrichment analysis, the number of pathways after enrichment of these genes was the same. As the DAU group, it was (Table 4) implied down-regulation of MF for immune response and up-regulation of MF for microtubule binding in the non-DAU group.

**Table 4:** Clusters of the PPI network of the DEGs in the DAU group

Cluster	Scores	Density	Nodes	Genes
1	24.917	25	299	GPSM1, ADRA2C, HTR1A, GALR2, BDKRB2, GNGT2, CXCR3, LPAR2, GRM3, TAS2R38, TAS1R1, PPY, TAS2R19, CXCL11, CXCL10, OXGR1, SST, SSTR4, GNGT1, GNG11, GNG13, TAS2R5, GNB4, GNB3, ADCY6
2	13.143	15	92	FBXO32, TRIM63, CCDC22, GPS1, UBE2C, TRAIP, FBXW12, DTX3L, FBXO27, SKP2, UBE2M, NEDD8, SKP1, RBX1, ASB12
3	11	11	55	GPR143, CHRM5, OPN4, KISS1, GPR132, P2RY2, F2RL2, CYSLTR2, GRP, GNRH2, PROKR2

The results of KEGG pathway enrichment analysis showed that DEGs in the DAU group were mainly enriched in pathogen infection signaling pathways and cancer-promoting signaling pathways, as well as in apoptosis and cell senescence. These pathways showed varying degrees of downregulation. Additional file 1: Figure S1 shows the intrinsic immune escape in the pathway, and the down-regulation of various related genes and proteins in the PI3K-Akt and mitochondrial pathways, with the ultimate biological effect being down-regulation of cellular senescence and apoptosis.

**Fig. 4:**
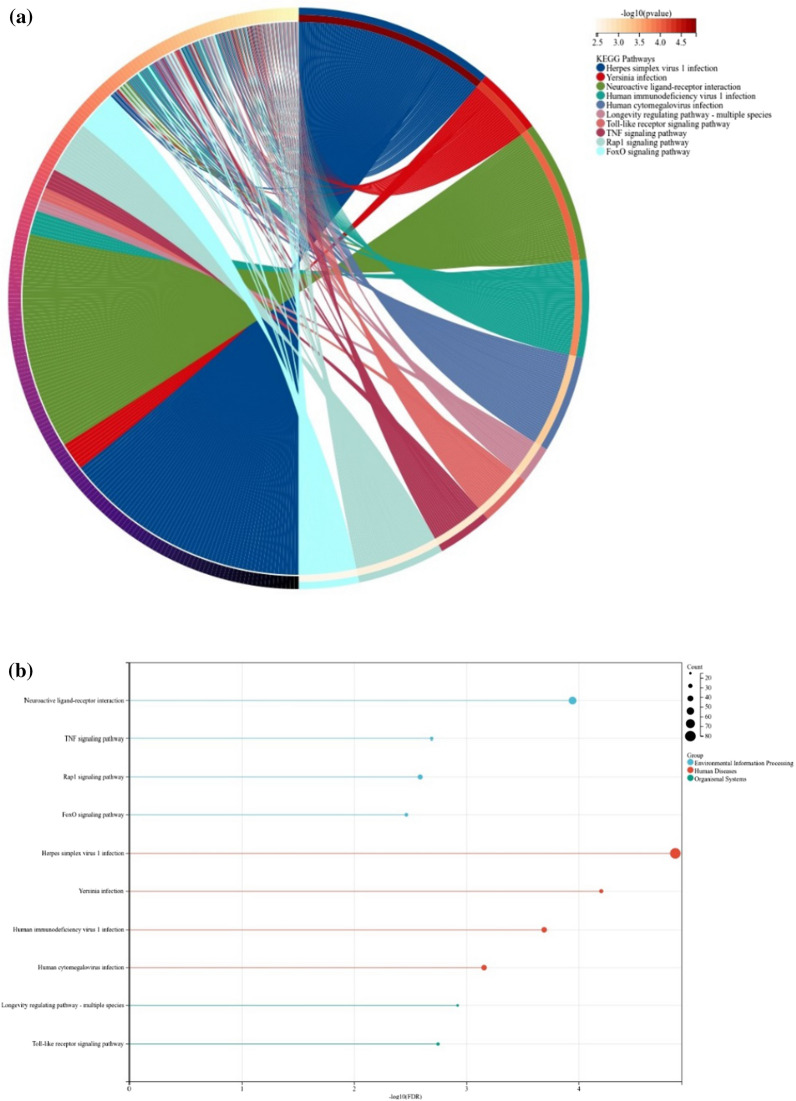
**a** Fig. 4a shows the chord diagram of the results of the KEGG pathway enrichment in the non-daily alcohol user group. In this figure, the following signaling pathways were enriched, HSV-1 infection, Yersinia infection, neuroactive ligand-receptor interaction , human immunodeficiency virus-1 infection, and human cytomegalovirus infection. **b** Fig. 4b shows the histogram of the results of the KEGG pathway enrichment in the non-daily alcohol user group

In addition, it can be obtained in the Fig. 4, the results of KEGG pathway enrichment analysis showed that DEGs in the non-DAU group were mainly enriched in pathogen infection signaling pathways, oxidative stress signaling pathways, and cell necrosis signaling pathways. These signaling pathways are all down-regulated to varying degrees. Additional file 1: Figure S2 shows the Yersinia infection pathway, and downregulation of this pathway inhibits the proliferation and recruitment of macrophages, impairs immune responses, and inhibits interferon responses, inflammatory responses, and phagocytosis.

### PPI network construction and WGCNA

Additional file 1: Figure S3 and Figure S4 show the PPI networks in the DAU and non-DAU groups, respectively.

Using the PPI network, we found three main clusters and there were PPIs in these three clusters, as we can see in the Table 4. Cluster 1 consisted of 25 proteins whose functions included chemotaxis and G protein-coupled receptor. Cluster 2 contained 15 proteins whose functions included cell cycle regulation, as well as regulation of calcium and NF-κB signaling pathways. Cluster 3 covered 11 proteins whose functions included G protein-coupled receptor, regulation of cell proliferation and apoptosis, and metastasis suppressor genes.

Similarly, we found two main clusters in the non-DAU group, which is shown in Table 5. Cluster 1 consisted of 24 proteins whose functions included chemotaxis of inflammatory cells and G protein-coupled receptor. Cluster 2 covered 20 proteins whose functions included cell cycle regulation, mitosis, and inflammatory response.

**Table 5:** Clusters of the PPI network of the DEGs in the non-DAU group

Cluster	Scores	Density	Nodes	Genes
1	14.696	24	169	ADRA2B, GNB3, GNB4, TAS2R3, GNG13, GNG11, GNGT1, GLP2R, GNG3, RAMP2, CCR9, GPR45, CCL28, RLN2, SUCNR1, MC1R, GPSM2, F2RL1, CHRM4, GNRH1, GAST, TRHR, LTB4R, TRH
2	9.579	20	91	B9D2, DSN1, CENPH, CLASP1, NUP85, CENPO, CENPT, NUP43, NUP107, BUB1B, ASB1, SMURF2, KLHL11, KBTBD7, FBXL4, FBXO32, CDC23, WWP1, UBE2D2, TRIM41

**Fig. 5:**
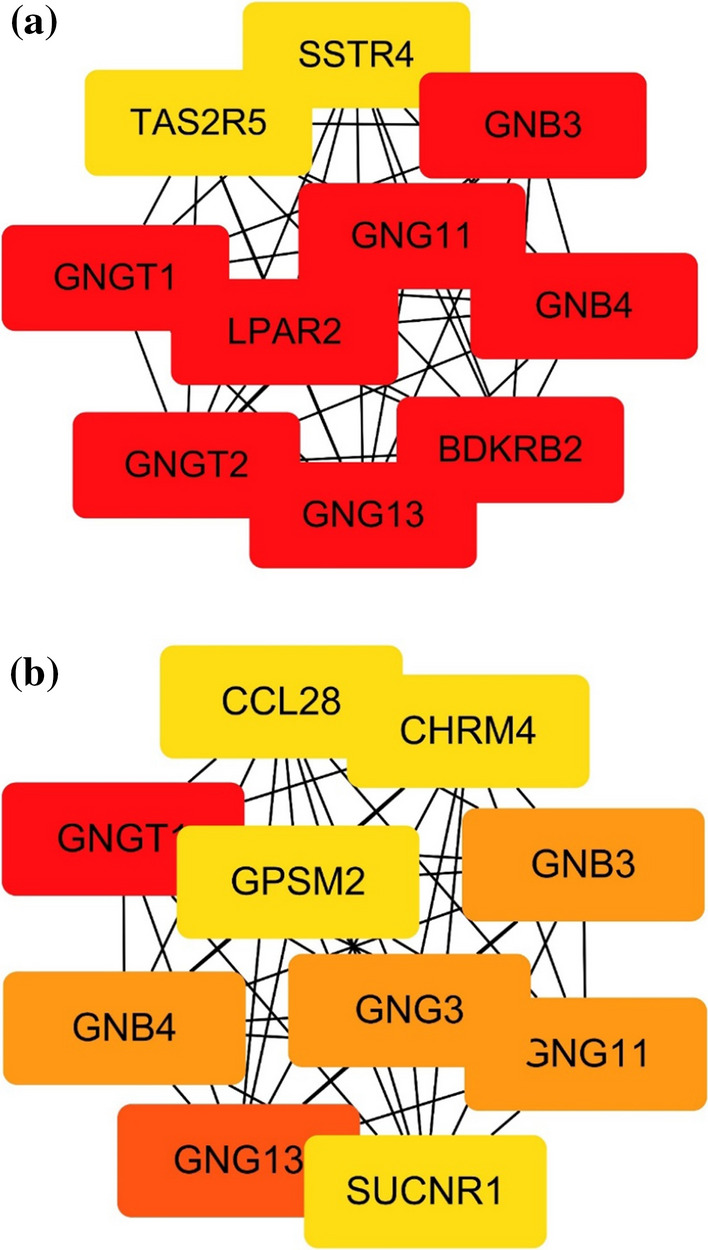
**a** Using the CytoHubba plugin of Cytoscape to screen the top 10 genes on the PPI network of the daily alcohol user group. The darker the color, the more important the gene is. **b** The top 10 genes were also screened for the PPI network of the non-daily alcohol user group. From the two figures, it can be found that there are differences between the two groups of hub genes

Using the MCODE, the most significant module, called sub-unit in the PPI networks, was identified. The hub genes and their corresponding lines in the two groups are shown in Fig. 5. The deeper color, the higher degree was.

**Fig. 6:**
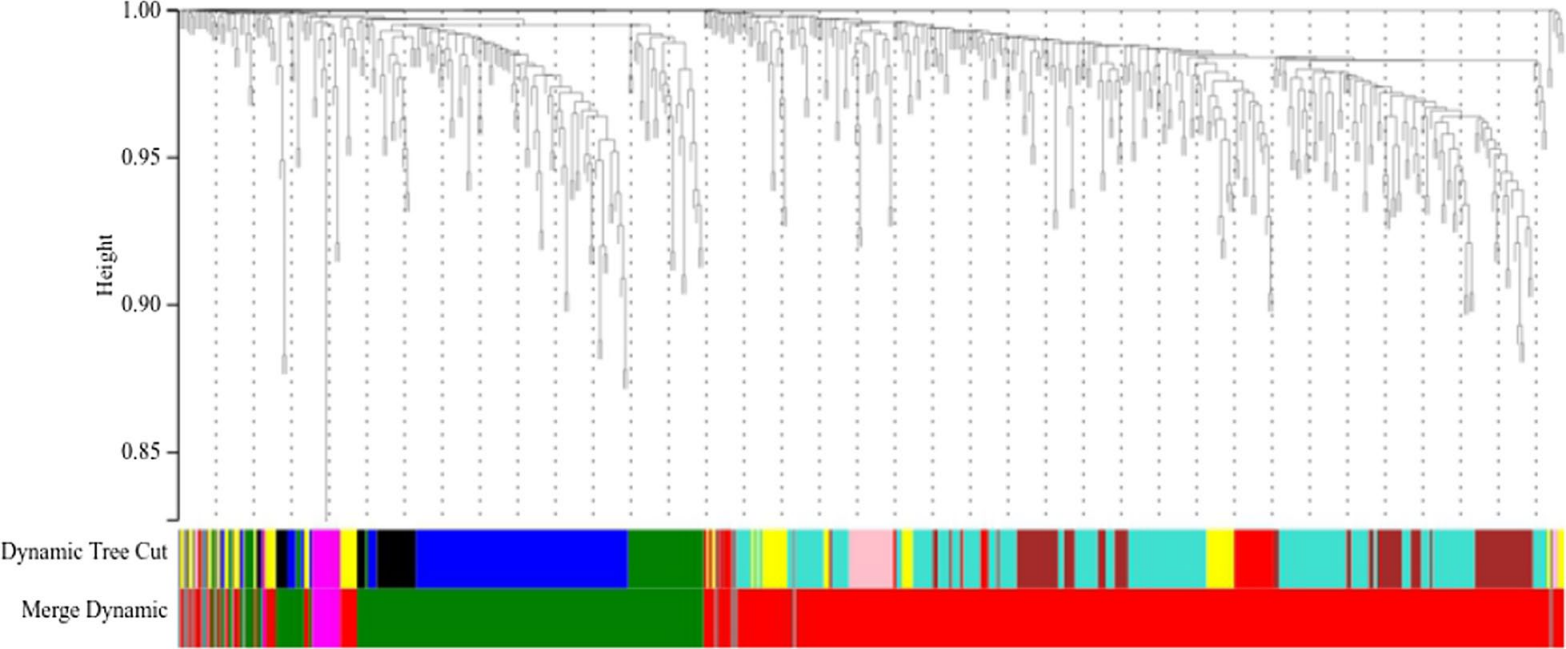
The WGCNA clustered the DEGs into 4 modules, and 4 different colors were used to represent 4 different modules

As shown in Fig. 6, the WGCNA clustered the DEGs into 4 modules, and 4 different colors were used to represent 4 different modules.

## Discussions

The results of the present study further indicated the molecular mechanism of Lp299v supplementation. Meanwhile, the hub genes and the results of enrichment analyses expanded the molecular mechanism of Lp299v supplementation in patients with stable CAD. Our experimental data were categorized into the DAU group and the non-DAU group. The former was composed of 8 samples, and the latter included 30 samples. The bioinformatics analysis was (Fig. 6) performed on both the DAU and the non-DAU groups. We found some differences between the results of two groups, which could reveal the weakening effect of alcohol on the protection of intestinal inflammation. This hypothesis will be further analyzed in the future experiments.

The composition of gut microbiota is very complex, including bacteroidetes, firmicutes, proteobacteria, fusobacteria, actinobacteria. In different populations with different diets, gut microbiota varies in terms of number and proportion (Yamashita et al. 2016). Previous studies have found that gut microbiota was associated with cardio-metabolic diseases (Zhou et al. 2020). Some scholars demonstrated that intestinal inflammation may be a risk factor for atherosclerosis, and gut microbiota and intestinal immunity could be used as therapeutic targets for the treatment of CAD (Yamashita et al. 2015). *Lactobacillus plantarum* is one of the important members of the genus Lactobacillus, and it has been identified as a probiotic, confirming its value for further research and application (Kleerebezem et al. 2010).

In both the DAU and non-DAU groups, the GO enrichment analysis indicated cell mitosis and reduced chemotactic movement, suggesting that Lp299v could promote immune regulation and attenuate immune response. Additionally, the GO enrichment analysis revealed that mitosis was up-regulated, apoptosis and cellular senescence were down-regulated, which may be secondary to the activation of the PI3K-Akt pathway. The KEGG pathway enrichment analysis showed apoptosis and senescence of cells in the DAU group, as well as the down-regulated expression of pathogen infection signaling pathways and cancer-promoting signaling pathways, as well as apoptosis and cellular senescence. While in the non-DAU group, pathogen infection signaling, oxidative stress signaling, and apoptosis signaling were down-regulated to varying degrees. In the DAU group, activation of these pathways inhibited cellular senescence and apoptosis, attenuated innate immune escape, and reduced inflammatory response. In the non-DAU group, activation of these pathways inhibited the proliferation and recruitment of macrophages, decreased the expression of interferon-β, and attenuated immune response and chemotactic cytokines. A previous study showed that alcohol intake could promote the growth of Gram-negative bacteria in the intestines and increase the permeability of the intestines, leading to systemic inflammations, which may explain the difference in the results of the KEGG analysis between the DAU and non-DAU groups (Parlesak et al. 2000). Another study showed that Lp299v reduced levels of IL-8, IL-12 and improved vascular endothelium, which supports our results (Malik et al. 2018). A number of scholars confirmed that Lp299v could attenuate the immune response in patients with coronary heart disease (Hofeld et al. 2021; Naruszewicz et al. 2002). A previous study reported that Lp299v significantly reduced cell apoptosis, which was consistent with the down-regulation of apoptosis pathway found in our study (Dykstra et al. 2011). Earlier studies showed that Lp299v-contained beverages could protect body cells against excessive production of reactive oxygen species, thereby protecting against oxidative damage (Gawlik-Dziki et al. 2021; Onning et al. 2003). These findings experimentally support our findings in the bioinformatics analysis.

We, in the present study, found genes that were associated with G protein-coupled receptors, immune responses, cell proliferation, and apoptosis regulation in the PPI networks. A previous study reported that Lp299v could be used in the treatment of some types of cancer, possibly because Lp299v promotes apoptosis, suppresses (Table 5) inflammation, and inhibits cell proliferation (Kazmierczak-Siedlecka et al. 2020). In our screening of hub genes, it was found that the majority of the hub genes were G protein-coupled receptor-related genes, followed by inflammation-activated chemotaxis-related genes.

Diverse types of chemokines act through G protein-coupled receptors, which are collectively known as chemokine receptors. Interleukin receptor and histamine receptor are involved in inflammation and allergic reactions. In the current study, it was hypothesized that one or more of the components of Lp299v could modulate the G protein-coupled receptor, thereby regulating the immune system, inhibiting inflammatory response, promoting cell mitosis and proliferation, and inhibiting cellular senescence and apoptosis.

We found the expressions of the genes encoding adenylate cyclase, and β- and γ-subunit of G protein-coupled receptor. The results of enriched MF of DEGs indicated that chemokine receptors, acting as a G protein-coupled receptor, could play important roles in the immune system cell signaling pathway. Using the constructed PPI networks, it was found that the expressions of CXCL11 and CXCR3 in the DEGs of this sample had a parallel relationship. Both CXCL11 and CXCR3 are pro-inflammatory factors. Hence, it was further hypothesized that Lp299v supplementation could modulate the expressions of chemokine receptors via a subsequent cellular effect.

In summary, Lp299v could treat patients with stable CAD by modulating inflammatory responses. Our study also found the role of G protein-coupled receptor, mitosis, apoptosis, and senescence of cell in the process of Lp299v supplementation. Cell mitosis, apoptosis, and senescence were associated with the PI3K-Akt pathway. The chemokine receptors could act as a G protein-coupled receptor, playing important roles in the immune system cell signaling pathway. It was supposed that chemokine receptors could have a cellular effect through the Gs-cAMP-PKA signaling pathway.

In this study, we found the following limitations. The small sample size might cause the bias in the experiment. We identified several hub genes and molecular pathways closely related to the effects of Lp299v supplementation, we didn’t explore the interactions between these hub genes yet. We used an unadjusted P-value < 0.05 as the threshold for significant difference and did not account for FDR, so the results of these analyses are purely exploratory.Thus, our findings remain to be further verified by additional in vitro experiments.

## Supplementary Information

**Additional file 1: Fig. S1.** Herpes simplex virus 1 infection-related pathway. **Fig. S2.** Yersinia infection-related pathway.** Fig. S3.** The PPI network of the DEGs in the daily alcohol user group. **Fig. S4.** The PPI network of the DEGs in the non-daily alcohol user group.** Fig. S5.** The WGCNA clustered the DEGs into 4 modules, and 4 different colors were used to represent 4 different modules. **Table S1.** The KEGG pathway enrichment analysis of DEGs in the daily alcohol user group sorted by adjusted P-values in a descending order. **Table S2.** The KEGG pathway enrichment analysis of DEGs in the non-daily alcohol user group sorted by adjusted P -values in a descending order.

## Data Availability

The datasets generated during and/or analyses during the current study are available in the GEO database (https://www.ncbi.nlm.nih.gov/geo/query/acc.cgi?acc=GSE156357).

## Abbreviations

CAD: Coronary atherosclerotic disease
Lp299v: *Lactobacillus plantarum* 299 V
GEO: Gene expression omnibus
GO: Gene ontology
KEGG: Kyoto encyclopedia of genes and genomes
PPI: Protein–protein interaction
DAVID: Database for annotation, visualization and integrated discovery
STRING: Search tool for the retrieval of interacting genes
DAU: Daily alcohol user
non-DAU: Non-daily alcohol user
FDR: False discovery rate
MCODE: Molecular complex detection
TOM: Topological overlap matrix
WGCNA: Weighted gene co-expression network analysis
CC: Cellular component
BP: Biological process
MF: Molecular function
