# Supplementary material for: Microarray analysis reveals the potential molecular mechanism of Lp299v in stable coronary atherosclerotic disease
Source: AMB Express. 2022 Sep 24;12:125. doi: 10.1186/s13568-022-01466-y (PMC9509519; doi:10.1186/s13568-022-01466-y)
Supplement: Supplementary file 1 — Additional file 1: Fig. S1. Herpes simplex virus 1 infection-related pathway. Fig. S2. Yersinia infection-related pathway. Fig. S3. The PPI network of the DEGs in the daily alcohol user group. Fig. S4. The PPI network of the DEGs in the non-daily alcohol user group. Fig. S5. The WGCNA clustered the DEGs into 4 modules, and 4 different colors were used to represent 4 different modules. Table S1. The KEGG pathway enrichment analysis of DEGs in the daily alcohol user group sorted by adjusted P-values in a descending order. Table S2. The KEGG pathway enrichment analysis of DEGs in the non-daily alcohol user group sorted by adjusted P -values in a descending order. [file 13568_2022_1466_MOESM1_ESM.docx]

Fig.S1

**
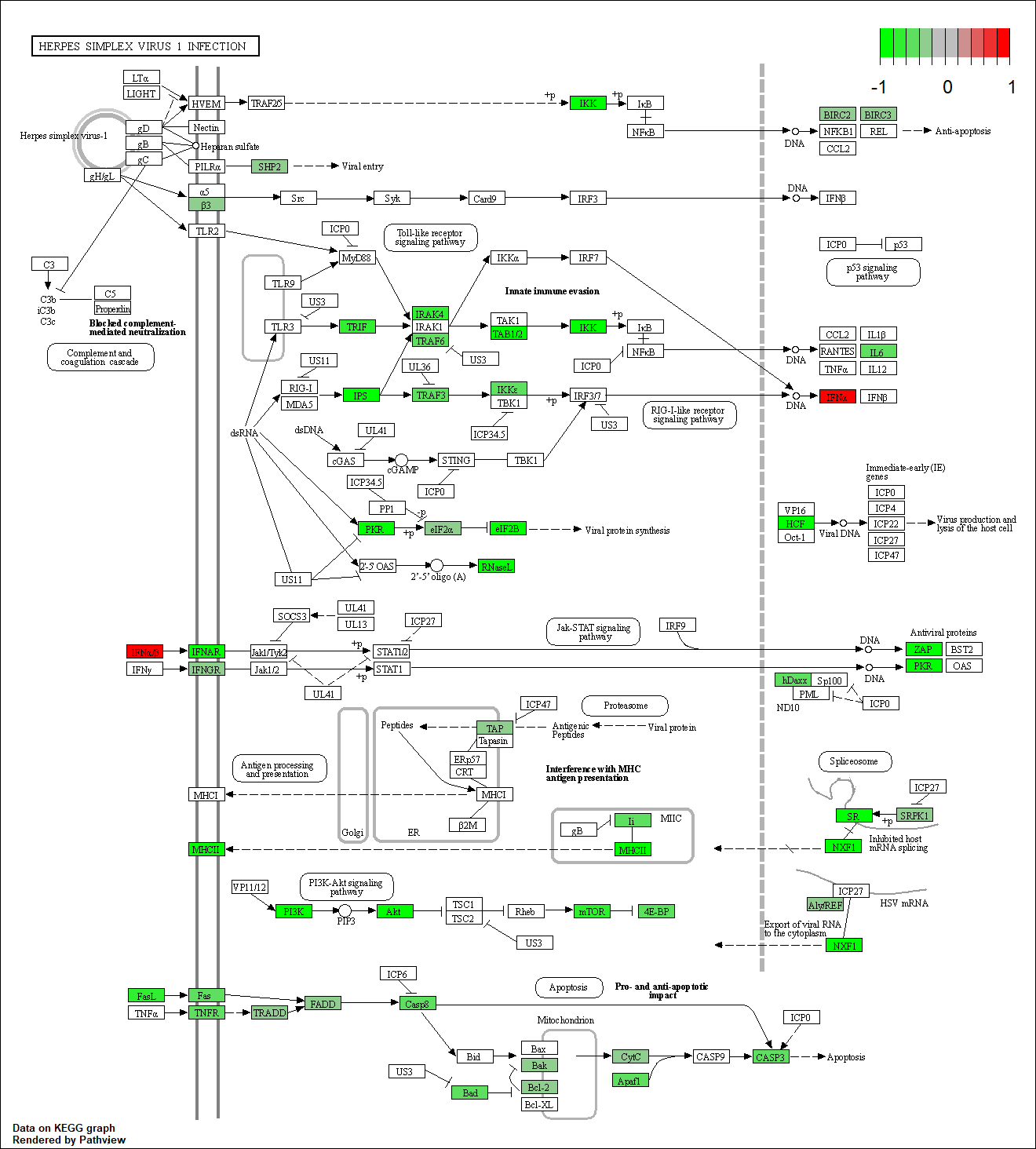
**

Herpes simplex virus 1 infection-related pathway.

Fig.S2

**
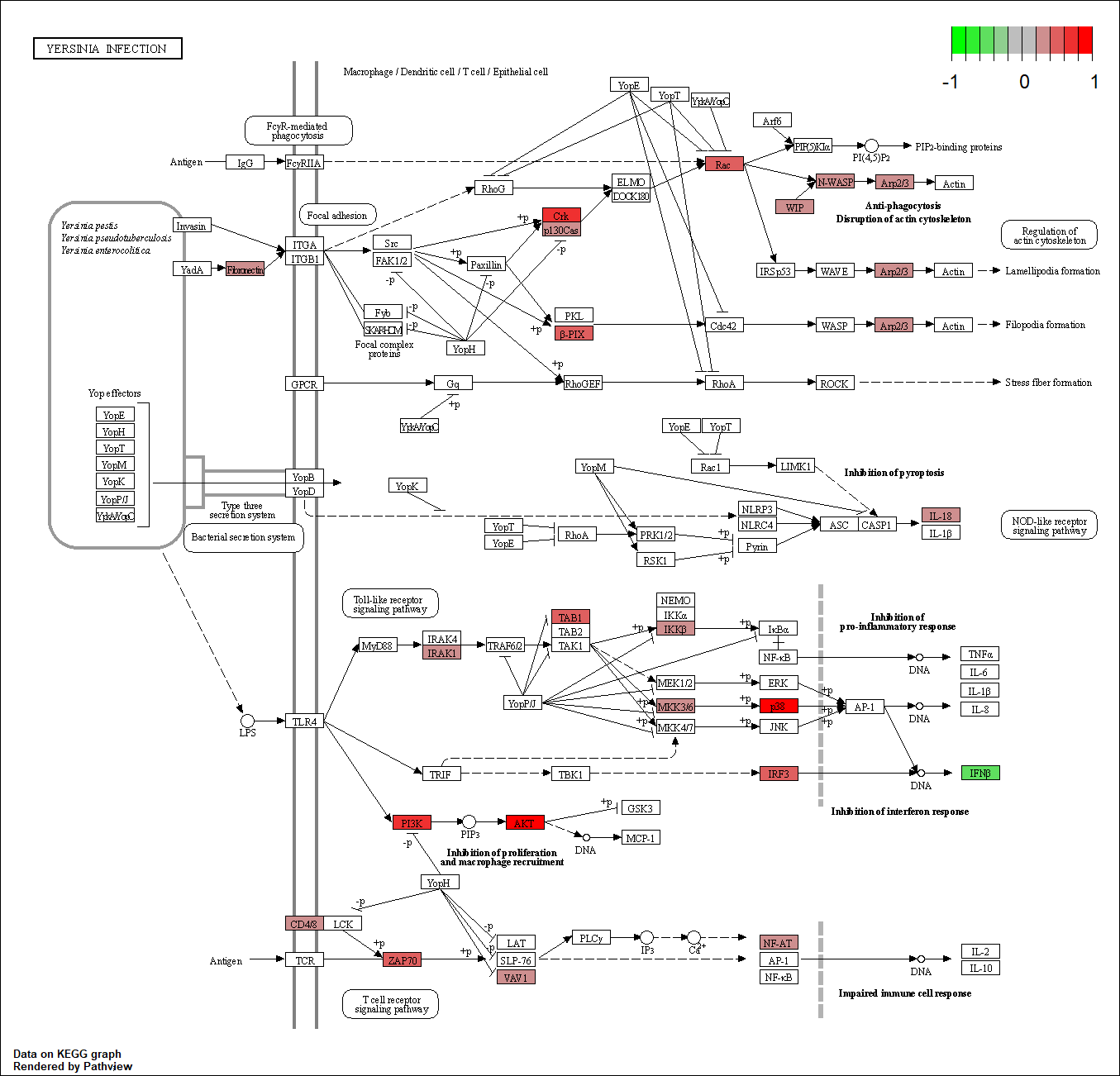
**

Yersinia infection-related pathway.

Fig.S3


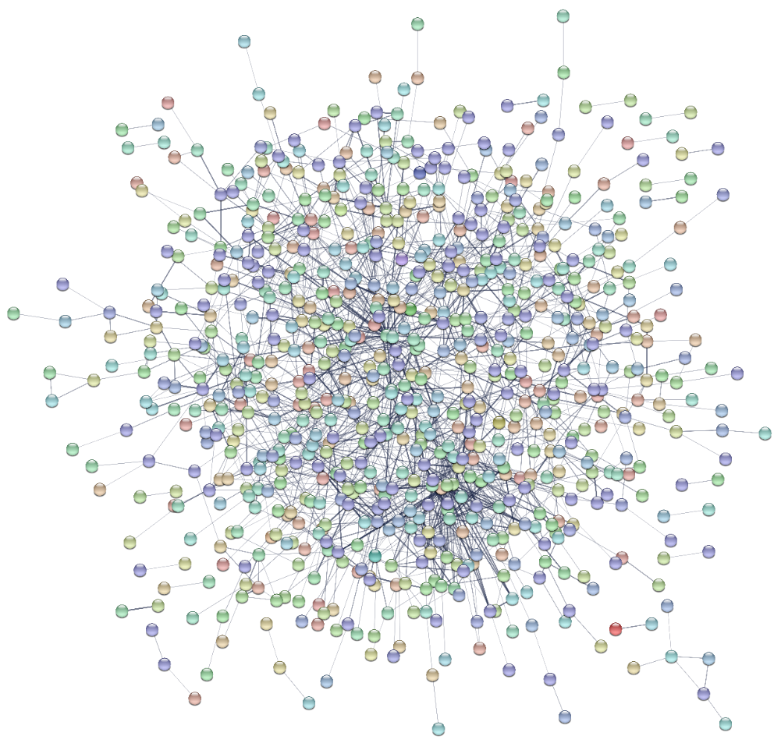


The PPI network of the DEGs in the daily alcohol user group.

Fig.S4


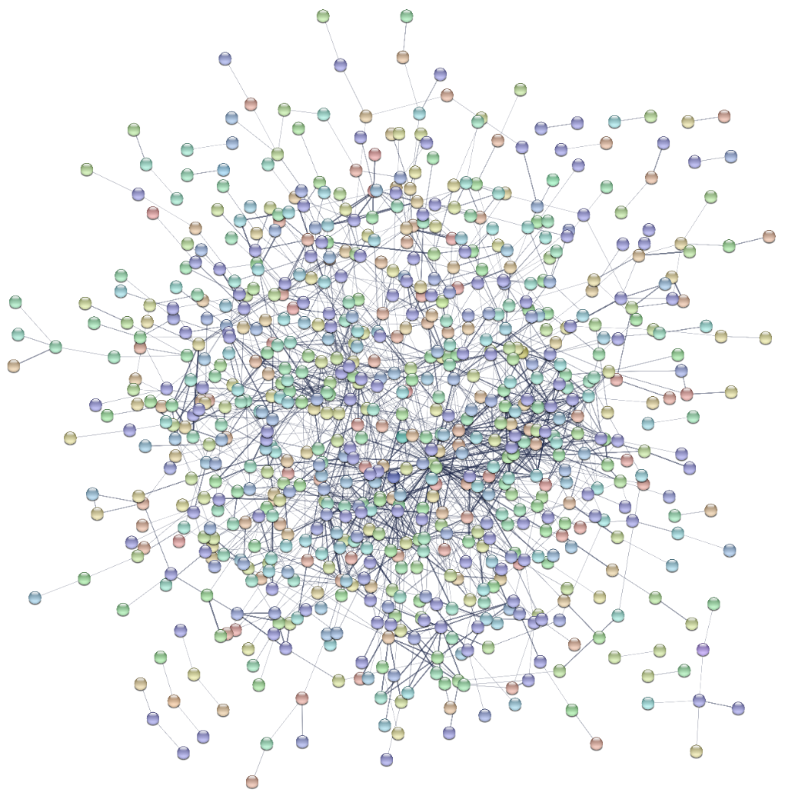


The PPI network of the DEGs in the non-daily alcohol user group.

Table S1

**The KEGG pathway enrichment analysis of DEGs in the daily alcohol user group sorted by adjusted *P*-values in a descending order**

| Term | Count | *Adjusted P* -value | *Regulation* |
| --- | --- | --- | --- |
| Herpes simplex virus 1 infection | 201 | 7.69E-05 | Down |
| GnRH secretion New! | 34 | 0.014111365 | Down |
| Ubiquitin mediated proteolysis | 62 | 0.014111365 | Up |
| Apoptosis | 61 | 0.020812592 | Down |
| Endocrine resistance | 46 | 0.023186664 | Down |
| Cellular senescence | 69 | 0.023186664 | Down |
| Glycosylphosphatidylinositol (GPI)-anchor biosynthesis | 16 | 0.025492935 | Down |
| Non-small cell lung cancer | 33 | 0.02752319 | Down |
| GnRH signaling pathway | 43 | 0.034087943 | Up |
| Colorectal cancer | 40 | 0.039585647 | Down |

Table S2

**The KEGG pathway enrichment analysis of DEGs in the non-daily alcohol user group sorted by adjusted** ***P* -values in a descending order.**

| Term | Count | Adjusted *P*-value | Regulation |
| --- | --- | --- | --- |
| Herpes simplex virus 1 infection | 80 | 0.00447729360856864 | Down |
| Yersinia infection | 27 | 0.0101657755294854 | Down |
| Neuroactive ligand-receptor interaction | 57 | 0.0122507189186799 | Up |
| Human immunodeficiency virus 1 infection | 39 | 0.0162558276352475 | Down |
| Human cytomegalovirus infection | 39 | 0.0449511423482168 | Down |
| Longevity regulating pathway - multiple species | 15 | 0.0646532693686372 | Up |
| Toll-like receptor signaling pathway | 21 | 0.081461013232851 | Down |
| TNF signaling pathway | 22 | 0.081461013232851 | Down |
| Rap1 signaling pathway | 35 | 0.091879038022896 | Up |
| FoxO signaling pathway | 24 | 0.100163950176909 | Down |
